# Selection tools and student diversity in health professions education: a multi-site study

**DOI:** 10.1007/s10459-022-10204-9

**Published:** 2023-01-18

**Authors:** S. Fikrat-Wevers, K. M. Stegers-Jager, P. M. Afonso, A. S. Koster, R. A. Van Gestel, M. Groenier, J. H. Ravesloot, A. Wouters, W. W. Van Den Broek, A. M. Woltman

**Affiliations:** 1 Institute of Medical Education Research Rotterdam, Erasmus MC, University Medical Center Rotterdam, Room AE-207, PO Box 2040, 3000 CA Rotterdam, The Netherlands; 2 Department of Biostatistics, Erasmus MC University Medical Center Rotterdam, Rotterdam, The Netherlands; 3 Department of Epidemiology, Erasmus MC University Medical Center Rotterdam, Rotterdam, The Netherlands; 4 Department of Pharmaceutical Sciences, Utrecht University, Utrecht, The Netherlands; 5 Technical Medical Centre, Technical Medicine, University of Twente, Enschede, The Netherlands; 6 Department of Medical Biology, Amsterdam UMC, University of Amsterdam, Amsterdam, The Netherlands; 7 Faculty of Medicine VU, Amsterdam UMC Location Vrije Universiteit Amsterdam, Boelelaan 1117, Amsterdam, The Netherlands; 8 LEARN! Research Institute for Learning and Education, Faculty of Psychology and Education, VU University Amsterdam, Amsterdam, The Netherlands

**Keywords:** Selection, Admission, Student diversity, Adverse impact, Undergraduate education

## Abstract

Student diversity in health professions education (HPE) can be affected by selection procedures. Little is known about how different selection tools impact student diversity across programs using different combinations of traditional and broadened selection criteria. The present multi-site study examined the chances in selection of subgroups of applicants to HPE undergraduate programs with distinctive selection procedures, and their performance on corresponding selection tools. Probability of selection of subgroups (based on gender, migration background, prior education, parental education) of applicants (N = 1935) to five selection procedures of corresponding Dutch HPE undergraduate programs was estimated using multilevel logistic regression. Multilevel linear regression was used to analyze performance on four tools: prior-education grade point average (pe-GPA), biomedical knowledge test, curriculum-sampling test, and curriculum vitae (CV). First-generation Western immigrants and applicants with a foreign education background were significantly less likely to be selected than applicants without a migration background and with pre-university education. These effects did not vary across programs. More variability in effects was found between different selection tools. Compared to women, men performed significantly poorer on CVs, while they had higher scores on biomedical knowledge tests. Applicants with a non-Western migration background scored lower on curriculum-sampling tests. First-generation Western immigrants had lower CV-scores. First-generation university applicants had significantly lower pe-GPAs. There was a variety in effects for applicants with different alternative forms of prior education. For curriculum-sampling tests and CVs, effects varied across programs. Our findings highlight the need for continuous evaluation, identifying best practices within existing tools, and applying alternative tools.

## Introduction

Medical schools and other health professions education (HPE) schools are responsible for selecting qualified students, as well as generating student populations that reflect the diverse society they will serve in the future (General Medical Council, 2015). A diverse healthcare workforce, aside from issues of equity and fairness, is important to improve the cultural competency of healthcare providers, and increase and equalize access to high-quality healthcare for different population groups (Cohen et al., 2002; Morgan et al., 2016). However, student diversity can be affected by the use of selection procedures for undergraduate HPE programs, as selection chances are unequally distributed across subgroups of applicants (Fielding et al., 2018; Mathers et al., 2016). Selection procedures cannot only include a great variety of tools, either defined at a national level or by an individual school, but the same tool can also be implemented in different ways. This raises the question if different tools have differential effects on student diversity (Patterson et al., 2016), and if some tools are more context-independent than others concerning their impact on student diversity. In the present multi-site study, we examined the selection chances of applicant subgroups and their performance on different selection tools in multiple contexts.

So far, literature has shown that selection procedures mainly negatively affect the selection chances of applicants with lower socio-economic status (SES) and from ethnic minorities (Fielding et al., 2018; Mathers et al., 2016; Mulder et al., 2022; Stegers-Jager et al., 2015; Steven et al., 2016). However, this effect may not always be straightforward, as performance differences between subgroups can depend on the combination of tools used in the procedures (Stegers-Jager, 2018). Traditionally, selection procedures in the United States and Europe mainly included prior education grade point average (pre-GPA) and cognitive tests, aimed at measuring intellectual ability. In the past decades, there has been a shift towards the inclusion of broadened selection criteria, which aim to add to the information derived from traditional tools, and often intend to evaluate personal qualities (Niessen & Meijer, 2017; Stegers-Jager, 2018). Examples include curriculum vitae (CV) and situational judgement tests (SJT). In this paper, we will refer to this distinction with the terms traditional and broadened criteria.

Prior research demonstrated performance discrepancies on traditional criteria, favoring higher SES and ethnic majority applicants (Girotti et al. 2020; Juster et al., 2019; Lievens et al., 2016; Stegers-Jager et al., 2015). Although broadened selection criteria were partly introduced to mitigate these adverse effects on student diversity, results so far are inconsistent (Stegers-Jager, 2018). For instance, Lievens et al (2016) found that the inclusion of an SJT in the United Kingdom could increase the representation of lower SES applicants, but not of ethnic minority applicants. However, a similar study in the United States found that adding an SJT was advantageous for the representation of both lower SES and ethnic minority applicants (Juster et al., 2019). This implies that the effects of selection tools on diversity can be context-dependent, at least in the case of broadened criteria. The curriculum-sampling test is another tool assessing broadened criteria that is increasingly used in international contexts and proved effective in terms of predicting academic achievement (Niessen et al., 2018). Curriculum-sampling tests mimic representative parts of a subject of the academic program. Generally, applicants study literature or watch video lectures from small-scale versions of an introductory course, followed by an exam (Niessen et al., 2018). Curriculum-sampling tests are aimed at measuring a mixture of attributes such as knowledge, motivation, and time spent studying (Niessen & Meijer, 2017). Additionally, these tests intend to assess the applicants’ ‘fit’ with the program (e.g., the way of testing and studying). To our knowledge, subgroup performance differences on this specific tool have not yet been investigated.

Every country has its own laws and regulations for selection and admission, as well as a unique context regarding student diversity. Typical for the Netherlands is that, after years of lottery, programs are now responsible for designing their own selection procedures. Programs independently decide which tools they include (both self-developed and standardized), and how many they include (with a minimum of two). This results in a great variety of procedures and tools. Results from a national retrospective study indicate that since the abolishment of lottery, inequality in selection chances between subgroups of applicants has increased (Mulder et al., 2022). The authors found that women, ethnic majority applicants, and applicants with higher SES had a higher probability of admission compared to their peers. However, this study did not take into account the role of the extensive range of possible selection procedures and tools. One previous single-site study attempted to unravel this matter, and concluded that ethnic minority and lower SES applicants had lower scores on academic criteria, but not on non-academic criteria (Stegers-Jager et al., 2015). The researchers discovered that for the institution under consideration, men had higher selection chances compared to women, which was again only related to performance on academic criteria. This contradicts the findings of the aforementioned national study (Mulder et al., 2022). This strengthens the hypothesis that the effects of selection on student diversity are context-dependent. An additional observation of the single-site study was that being a first-generation immigrant was correlated with poorer selection outcomes (Stegers-Jager et al., 2015), a variable that was not accounted for in the national cohort study. A final potentially relevant variable that was included in neither of the studies, is prior education. A recent report indicated that applicants with prior foreign education had smaller selection chances compared to applicants from the ‘traditional’ pre-university track (Van Den Broek et al., 2018).

In short, it is not clear how different selection tools can affect student diversity across different contexts. The freedom of Dutch HPE programs to design their selection procedures creates the unique opportunity to compare the effects of selection on student diversity across different procedures with a variety of selection tools. The present prospective multi-site study aimed to evaluate the probability of selection into five undergraduate HPE programs for subgroups of applicants based on gender, migration background (as an indicator of ethnicity), parental education (as an indicator of SES), and prior education. Additionally, we examined performance differences on two traditional selection tools (pre-GPA, biomedical knowledge test), and two tools assessing broadened criteria (curriculum-sampling test, CV).

## Method

### Design and context

The present research concerns a prospective multi-site cohort study. We collected data from five university-level undergraduate HPE programs in the Netherlands, including three medical programs (labeled A, B, and C), one technical-medical (clinical technology) program (labeled D), and one pharmacy program (labeled E). The included programs were located in different parts of the Netherlands, both in urban and rural areas, and were all concerned about enhancing diversity in their selection processes.

Uniquely to the Netherlands is that admission requirements of different types of undergraduate HPE programs are identical. To be eligible, applicants need to meet the same stringent requirements regarding subjects taken (e.g., physics, chemistry, and biology) and educational level. Consequently, the applicant pools are relatively homogeneous in terms of academic background; students who apply to a university-level undergraduate HPE program are already strongly preselected based on academic skills due to highly selective secondary education (Niessen & Meijer, 2016). When applicants apply to their program of choice, they apply to one specific institution. Each institution has a predetermined fixed number of spots. By law, institutions are required to include at least two selection criteria, but as previously mentioned, there are no additional requirements concerning, for instance, the content and quality of the tools. Consequently, great variety exists in the selection procedures that programs employ, both between and within different types of HPE programs at different institutions. We studied tools used by more than one program, to evaluate whether effects were similar or different across programs.

The selection procedures of the five programs are described in Table 1. The tools used by multiple programs were pre-GPA, biomedical knowledge test, curriculum-sampling test, and CV. Pre-GPA comprised of applicants’ average school grades on required subjects, usually mathematics, physics, biology, and/or chemistry. Biomedical knowledge tests assessed applicants’ existing general knowledge about biomedical subjects, without requiring any preparation. Curriculum-sampling tests were (largely) based on preparatory materials in the form of a lecture and/or reading materials that applicants had received some weeks prior to the testing day. CVs consisted of an assessment of extracurricular activities, such as (voluntary) jobs, internships, or evidence of extraordinary cultural or athletic skills. One standardized tool was included, the biomedical knowledge subtest of the BioMedical Admissions Test (BMAT), which was administered by one program (D). All other selection tools in our sample were self-developed by the individual programs. Consequently, the specific application of the tools differed between the programs, e.g., the specific subjects included in pre-GPA and the types of questions in tests. All selection tools, except for the BMAT, were administered in Dutch. Programs were responsible for their own quality assurance, and we did not have access to psychometric information.

**Table 1:** Selection procedures of the five programs included in the present study

Program^a^	Track	Type of program	Procedure	Weighting (%)
A	A	Medicine	Procedure included only an on-site testing day with the following tests	
			Curriculum-sampling test.^*^ Multiple-choice test based on online study materials (texts, video lecture, knowledge clips)	50
			Biomedical knowledge test.^*^ Multiple-choice test on the application of biomedical knowledge typically covered in pre-university education, and on news topics in the medical field	50
			*Medical ethical essay test.* Writing task of three short essays to reflect on medical ethical dilemmas. This test only counted for 150 applicants with an identical sum score on the other two tests and scoring around the threshold for these tests	0/decisive in the event of a tie
B	B	Medicine	Procedure included an entry form including the following	
			Pre-GPA.^*^ Based on the grades for Dutch and English language, mathematics, physics, science, and biology. For applicants not applying during their final year of pre-university education (i.e., who graduated in a previous year or applied from an alternative type of education), pu-GPA wasn’t included and the other two criteria both weighted 50%	33
			CV.^*^ Included work experience, board position, or exceptional performance in the areas of science, arts, literature or sports. Applicants were assessed based on their argumentation and not the activity itself	33
			Normally, the procedure included an on-site testing day with multiple tests, but due to COVID-19-related measures, the testing day was off-site via online proctoring, including the following test:	
			Curriculum-sampling test.^*^ Test including both multiple choice and open items, based on a lecture, including mathematical items	33
C	C1 (70% of spots)	Medicine	This track included all applicants who were applying during their final year of pre-university education	
			*Round 1* included an entry form. A composite score was calculated on:	100
			*Prior education.* Scores based on grades and potential additional extracurricular education	
			CV.^*^ Either voluntary experiences or paid work in health care or community service, in leadership/organizational or entrepreneurial roles, in the areas of science or technology, or experiences demonstrating excellence in sports or the arts	
			Candidates with sufficiently high scores were invited for round 2	
			*Round 2* normally included a set of tests, but due to COVID-19-related measures these could not be administered. Therefore, the second-round final ranking was based on the composite score of round 1 with additional sub-ranking on the following criterium:	
			Pre-GPA.^*^ Based on grades for the science subjects	
C	C2 (30% of spots)	Medicine	This track included all applicants who were not applying during their final year of pre-university secondary education	
			*Round 1 and 2*. The same instruments as in track C1 were included, though the scoring method differed on some aspects to allow for differences in prior education paths, e.g., the inclusion of a sub-score for tertiary education performance	
D	D	Technical medicine	Procedure included an entry form including the following	
			Pre-GPA.^*^ Based on the grades for mathematics, physics, biology, and overall pu-GPA	60
			The BMAT was administered during an on-site testing day	
			*Aptitude and skills test (BMAT 1).* Multiple-choice test on general skills in problem solving, inferences, and data analysis	10
			Biomedical knowledge test (BMAT 2).^*^ Multiple-choice test on applying scientific knowledge from biomedical subjects covered in pre-university education	20
			*Writing task (BMAT 3).* Task on the ability to develop and organize ideas, and to communicate them in writing	10
E	E	Pharmacy	Procedure included an entry form including the following	
			Pre-GPA.^*^ Based on the grades for mathematics, physics, biology, and overall pu-GPA	40
			The following tests were administered during an on-site testing day	
			Curriculum-sampling test.^*^ Open questions test based on a bundle of articles, including mathematical items	30
			*Curriculum-sampling test (short-term).* Open questions test based on a lecture and problem-based learning tutorial that took place during the on-site testing day	30

Pre-GPA = prior education grade point average; CV = curriculum vitae; BMAT = BioMedical Admissions Test

^a^A = Amsterdam UMC, location AMC; B = Erasmus MC; C = Amsterdam UMC, location VUmc; D = University of Twente; E = Utrecht University

^*^Selection tools with an asterisk  were included in the multilevel linear regression analyses

### Participants and procedure

All applicants engaged in the selection procedures for entry in September 2020 (N = 3280) were invited to participate. For programs A, D, and E, applicants were invited during the on-site testing days. Programs B and C did not perform on-site testing due to COVID-19 pandemic measures, necessitating recruitment via e-mail during the selection procedure.

Applicants were requested to complete a demographics questionnaire. In this survey, applicants were asked to report their student number, gender, migration background, parental education and prior education. Data on performance on the selection procedures were derived from the related university student administration systems. Student number was used to connect the data from the demographics questionnaire with performance data.

Informed consent was obtained from all participants. Applicants were informed that participation was voluntary and would not influence their selection outcomes, and we made explicit that the researchers operated independently from the selection committees. Applicants did not receive incentives for participation in the study. All data were pseudonymized immediately after the demographics and performance data were combined. The Medical Ethical Review Committee of Erasmus MC declared the study exempt from ethical approval.

### Variables

Predictors included gender, migration background, prior education, and parental education.

Gender diversity was acknowledged in the present study, and applicants had the option to choose between three categories: ‘man’, ‘woman’, and ‘other, namely [free text box]’.

Migration background was used as a proxy for ethnicity, recognizing that this does not completely capture the multidimensional character of ethnicity. Migration background was defined in alignment with Statistics Netherlands (CBS). Individuals have a migration background when at least one of their parents was born outside of the Netherlands. Based on the taxonomy of CBS, we distinguished between a Western and non-Western migration background. All European (excluding Turkey), North American, and Oceanian countries, Indonesia, and Japan were considered Western. Non-Western countries included all countries in Africa, Asia (excluding Indonesia and Japan), Latin America, and Turkey. Additionally, we distinguished between first-generation and second-generation immigrants. First-generation immigrants were born outside the Netherlands. In the Netherlands, the use of migration background and CBS taxonomy are considered the standard for operationalizing ethnicity, also in research in HPE (e.g., Mulder et al., 2022; Stegers-Jager et al., 2015).

In the Netherlands, the typical educational route to an HPE program is the pre-university track of secondary school with a health/science profile. However, applicants can apply to HPE programs from alternative forms of prior education. We distinguished between standard Dutch pre-university education, university, higher vocational education, all forms of foreign education, and other forms of prior education (e.g., entrance exams and adult education).

Finally, parental education was used as a proxy for SES, acknowledging that this is only one of the many indicators that can be used to operationalize SES. Parental education was determined by the educational level of applicants’ parents. Applicants were categorized as first-generation university applicants when none of their parents had attended higher education, i.e., university or higher vocational education. First-generation university applicants were a subgroup of interest, because previous research has demonstrated that their odds of being selected into medical school are lower (Mason et al., 2021; Stegers-Jager et al., 2015). Additionally, they face numerous obstacles when applying to medical school, including a lack of knowledge about the admission process and financial barriers (Romero et al., 2020).

### Outcome measures

Five outcome measures were included. The first—binary—outcome measure indicated whether an applicant was selected (yes/no), determined by their ranking number. The other four outcome measures were continuous and reflected performance on the four tools: pre-GPA, curriculum-sampling test, biomedical knowledge test, and CV. For each tool, the responsible program calculated a raw score based on its own scoring method. Subsequently, each program transformed these raw scores into standardized Z-scores to enable comparisons of tools between tracks. These Z-scores were made available to the researchers and used for the analyses.

### Statistical analyses

Multilevel logistic regression analysis was performed to calculate odds ratios (OR) for the effect of the different predictors on the probability of selection. An OR of > 1 indicates an increased likelihood of selection. Since the content of the selection procedure differed between programs, we used the program to which the applicants applied as a random intercept in this model. Program E was excluded from this analysis, given the high selection rate of 96%, which was in large contrast with other programs having an average selection rate of 47% (Appendix 1: Table 6). The selection rate of this program was this high due to the small number of applicants compared to the number of available spots.

To compare performance on different tools, we used Z-scores that were provided by the participating programs. We performed multilevel linear regression to assess performance differences between Z-scores on the four overlapping tools. Program C applied different scoring methods for two independent selection tracks with intake restriction (Table 1), resulting in Z-scores for the two different tracks. Therefore, we used the variable ‘track’ instead of ‘program’ as a random intercept for the analyses of the four tools. This random effect was included because, as mentioned earlier, the specific application of each tool differed across settings.

We applied likelihood ratio tests with the boundary correction to assess whether the inclusion of ‘program’ or ‘track’ as a random intercept explained significantly more variance compared to the model without the intercept.

Analyses were executed using the LME4 1.1.26 and NLME 3.1.152 packages in R version 4.0.4. For all statistical analyses, assumptions were checked. We interpreted OR of > 1.68 or < 0.60 as a small effect, OR of > 3.47 or < 0.29 as a medium effect, and OR of > 6.71 or < 0.15 as a large effect (Chen et al., 2010).

## Results

### Applicant characteristics

In total, 1935 applicants participated in the study (response rate 59%, range 34–81% for individual programs). With respect to gender, 30% of the respondents identified as men, and one applicant identified as ‘other’. This individual was excluded from the subgroup analyses, and therefore only the categories of men and women are described in the results. Furthermore, 38% had a migration background, 20% applied from alternative forms of prior education, and 25% were first-generation university applicants. In terms of gender and age, all samples were representative of the complete applicant pool. For two programs (C and E), participating applicants performed slightly better on the selection (in terms of ranking number) compared to non-participating applicants.

Since applicants were exposed to some overlapping tools, but also to some unique tools, and one program was excluded from the analyses on the probability of selection, the distribution of applicant characteristics differed between the multilevel analyses (Table 2). Noteworthy is that for the biomedical knowledge test, the proportion of applicants with a migration background was relatively low compared to the other programs (28% vs 36–43%). Additionally, for pre-GPA, the proportion of applicants from alternative forms of prior education was comparatively small (10% vs 19–23%). This is probably caused by the fact that pre-GPA is not always included as a selection tool for those applicants.

**Table 2:** Applicant characteristics of the total sample and of the different multilevel analyses

	Total sample		Probability of selection (program A, B, C, D)		Performance on pre-GPA (track B, C1, C2, D, E)		Performance on biomedical knowledge test (track A, D)		Performance on curriculum-sampling test (track A, B, E)		Performance on CV (Track B, C1, C2)	
	N	%	N	%	N	%	N	%	N	%	N	%
Total	1935	100	1688	100	1130	100	743	100	1309	100	863	100
Gender												
Woman	1347	70	1168	69	803	71	494	66	899	69	617	71
Man	587	30	520	31	327	29	249	34	410	31	246	29
Migration background												
No migration background	1201	62	1080	64	719	64	531	71	766	59	493	57
1st generation Western background	45	2	43	3	17	2	9	1	30	2	31	4
2nd generation Western background	130	7	121	7	72	6	18	2	86	7	64	7
1st generation non-Western background	108	6	75	4	46	4	52	7	78	6	53	6
2nd generation non-Western background	452	23	367	22	275	24	134	18	349	27	221	26
Prior education												
Pre-university education	1543	80	1341	80	1012	90	600	81	1033	79	665	77
University	236	12	225	13	83	7	110	15	159	12	114	13
Higher vocational education	68	4	54	3	14	1	21	3	54	4	33	4
Foreign education	54	3	50	3	12	1	6	< 1	39	3	41	5
Other	32	2	17	1	9	1	6	< 1	24	2	10	1
Parental education												
No 1st generation university applicant	1445	75	1270	75	841	74	587	80	970	74	618	72
1st generation university applicant	483	25	412	25	289	26	156	20	339	26	245	28

N = number of individuals**;** pre-GPA = prior education grade point average; CV = curriculum vitae

The individual programs differed in their distribution of the applicant characteristics of interest (Appendix 1: Tables 6, 7). The most notable difference is that compared to the other programs, Program D—the only rural program in the sample—had a lower representation of applicants with a migration background (13% vs 32–53%) and first-generation university applicants (15% vs 24–32%). Differences in demographic composition are not caused by differences in admission requirements, since these were all comparable across programs. However, it is possible that other institutional-related factors made certain programs more attractive to specific subgroups, including location and selection procedure (Wouters et al., 2017b).

**Table 3:** Results multilevel logistic regression analysis for probability of selection (N = 1688)

Predictor	Applicant pool		Selected applicants		% Selected	Adjusted OR	95% CI
	N	%	N	%			
Intercept						1.07	0.92, 1.24
Gender							
Woman	1168	69	555	70	48	1	
Man	520	31	233	30	45	0.92	0.75, 1.14
Migration background							
No migration background	1080	64	530	67	49	1	
1st generation Western background	43	3	10	1	23	0.45*	0.20, 0.99
2nd generation Western background	121	7	62	8	51	1.12	0.76, 1.64
1st generation non-Western background	75	4	29	4	39	0.73	0.45, 1.20
2nd generation non-Western background	367	22	157	20	43	0.80	0.63, 1.02
Prior education							
Pre-university education	1341	80	650	82	49	1	
University	225	13	105	13	47	0.96	0.72, 1,28
Higher vocational education	54	3	19	2	35	0.60	0.34, 1.07
Foreign education^a^	50	3	12	2	24	0.46*	0.22, 0,94
Other	17	1	3	< 1	18	0.28	0.08, 1.00
Parental education							
No 1st generation university applicant	1270	76	612	78	48	1	
1st generation university applicant	412	25	177	22	43	0.85	0.68, 1.07

N = number of individuals. Adjusted OR refers to the adjusted odds ratio together with the 95% confidence interval (95% CI)

^a^Number of applicants with foreign education and % selected: no migration background (N = 9, 67%); first-generation western background (N = 22, 18%); second-generation western background (N = 8, 25%); first-generation non-western background (N = 7, 0%); second-generation non-western background (N = 4, 0%)

*p < .05

### Probability of selection

First-generation Western immigrants were significantly less likely to be selected compared to applicants without a migration background (23% vs 49%), corresponding to an adjusted OR of 0.45 (95% confidence interval [CI] [0.20, 0.99]; Table 3). Additionally, foreign-educated applicants had smaller selection odds than those from standard pre-university education (24% vs 49%, adjusted OR = 0.46, 95% CI [0.22, 0.94]). Both can be interpreted as small effects (i.e., OR < 0.60). The category ‘other forms of prior education’ demonstrated a medium (i.e., OR < 0.29), but non-significant (level 0.05) negative effect (18% vs 49%, adjusted OR = 0.28, 95% CI [0.08, 1.00]), which could be due to the small size of this group (N = 17). Gender and parental education were not significantly associated with the probability of selection. The random effect of program was not significant (SD = 0.00, 95% CI [0.00, 0.21], *p* = 0.50), indicating that subgroup differences in the probability of selection were similar across programs, given the fixed structure considered (i.e., the variables of gender, migration background, prior education and parental education).

### Performance on traditional criteria

#### Pre-GPA

Pre-GPA was used by four programs (B, C, D, and E), of which one program used two independent selection tracks, resulting in five tracks in the analysis (B, C1, C2, D, and E). Compared to traditional applicants, first-generation university applicants had significantly lower pre-GPAs (*B* = − 0.17, 95% CI [− 0.30, − 0.03]; Tables 4, 5). As Z-scores were used for all criteria, the unstandardized Bs indicate the difference in SD. Thus, for example, pre-GPAs of first-generation university applicants were 0.17 SD lower than those of non-first-generation university applicants. Applicants with university-level and with ‘other forms of prior education’ had significantly lower pre-GPA (respectively, *B* = − 0.41, 95% CI [− 0.63, − 0.18]; *B* = − 0.76, 95% CI [− 1.42, − 0.11] compared to standard pre-university applicants, while pre-GPAs of applicants with foreign education were significantly higher (*B* = 1.13, 95% CI [0.54, 1.72]). Gender and migration background were not associated with pre-GPA. The random effect of track was not significant (SD = 0.005, 95% CI [0, 506553], *p* = 0.50), indicating that the performance differences found on pre-GPA were similar across tracks, given the fixed structure considered.

**Table 4:** Descriptive statistics of subgroup performance on four selection tools

	Z-scores on pre-GPA (track B, C1, C2, D, E)			Z-scores on biomedical knowledge test (track A, D)			Z-scores on curriculum-sampling test (track A, B, E)			Z-scores on CV (Track B, C1, C2)		
	M	SD	95% CI	M	SD	95% CI	M	SD	95% CI	M	SD	95% CI
Gender												
Woman	0.03	0.99	− 0.04, 0.10	− 0.04	0.96	− 0.13, 0.04	0.03	0.93	− 0.03, 0.09	0.13	1.00	0.05, 0.21
Man	0.01	1.03	− 0.10, 0.12	0.15	1.05	0.02, 0.26	− 0.01	1.06	− 0.11, 0.09	− 0.04	0.99	− 0.16, 0.09
Migration background												
No migration background	0.05	0.98	− 0.02, 0.12	0.06	1.01	− 0.03, 0.14	0.12	0.91	0.05, 0.18	0.13	0.97	0.05, 0.22
1st generation Western background	0.33	11.53	− 0.5, 1.12	− 0.10	1.38	− 1.16, 0.96	− 0.15	0.98	0.52, 0.22	− 0.61	0.81	− 0.91, 0.32
2nd generation Western background	0.09	0.93	− 0.12, 0.31	− 0.04	0.97	− 0.31, 0.23	0.14	1.02	− 0.08, 0.35	0.05	1.02	− 0.21, 0.30
1st generation non-Western background	0.14	1.42	− 0.29, 0.56	− 0.14	0.91	− 0.59, 0.31	− 0.38	1.09	− 0.63, 0.14	0.01	1.14	− 0.30, 0.33
2nd generation non-Western background	− 0.11	0.96	− 0.22, 0.00	− 0.06	0.93	− 0.22, 0.10	− 0.12	1.03	− 0.23, − 0,02	0.11	1.02	− 0.03, 0.24
Prior education												
Pre-university education	0.05	1.00	− 0.01, 0.11	− 0.02	0.99	− 0.10, 0.06	0.01	0.96	− 0.05, 0.07	0.16	0.99	0.09, 0.24
University	− 0.36	0.73	− 0.52, 0.20	0.27	1.03	0.08, 0.46	0.36	0.80	0.23, 0.48	0.09	1.02	− 0.10, 0.28
Higher vocational education	− 0.20	0.66	− 0.59, 0.18	− 0.15	0.77	− 0.51, 0.20	− 0.22	1.26	− 0.56, 0.13	− 0.41	0.81	− 0.70, − 0.12
Foreign education	1.28	1.90	0.07, 2.49	− 0.16	1.39	− 1.44, 1.13	− 0.53	1.08	− 0.88, − 0.19	− 0.58	0.97	− 0.89, − 0.27
Other	− 0.75	0.68	− 1.27, − 0.23	0.28	1.11	− 0.89, 1.45	− 0.49	0.73	− 0.80, − 0.19	− 0.61	0.76	− 1.12, − 0.11
Parental education												
No 1st generation university applicant	0.08	1.02	0.01, 0.15	0.05	1.00	− 0.03, 0.13	0.05	0.94	− 0.01, 0.11	0.10	1.01	0.02, 0.18
1st generation university applicant	− 0.14	0.95	− 0.25, 0.03	− 0.07	0.96	− 0.21, 0.08	− 0.07	1.06	− 0.18, 0.05	0.04	1.04	− 0.08, 0.17

Pre-GPA = prior education grade point average; CV = curriculum vitae; M = mean; SD = standard deviation; 95% CI = 95% confidence interval. Although we use Z-scores, the overall mean can deviate slightly from 0, and the overall SD can deviate slightly from 1, because we only used data from applicants who provided consent, thus not all selection outcomes, on which the Z-scores are based, are included

**Table 5:** Results multilevel linear regression analyses for performance on four selection tools

	Z-scores on pre-GPA (N = 1130; track B, C1, C2, D, E)		Z-scores on biomedical knowledge test (N = 743; track A, D)		Z-scores on curriculum-sampling test (N = 1309; track A, B, E)		Z-scores on CV (N = 863; track B, C1, C2)	
	*B*	95% CI	*B*	95% CI	*B*	95% CI	*B*	95% CI
Intercept	0.12**	0.03, 0.21	− 0.04	− 0.14, 0.07	0.12	− 0.02, 0.26	0.26	− 0.05, 0.57
Gender								
Man	− 0.04	− 0.17, 0.09	0.21**	0.06, 0.37	− 0.03	− 0.14, 0.09	− 0.17*	− 0.31, − 0.02
Migration background								
1st generation Western background	0.06	− 0.44, 0.56	− 0.10	− 0.79, 0.60	0.00	− 0.39, 0.40	− 0.43*	− 0.85, − 0.00
2nd generation Western background	0.05	− 0.19, 0.30	− 0.09	− 0.37, 0.20	0.09	− 0.13, 0.30	− 0.02	− 0.28, 0.24
1st generation non-Western background	0.12	− 0.18, 0.42	− 0.30	− 0.77, 0.17	− 0.43**	− 0.67, − 0.20	− 0.03	− 0.32, 0.25
2nd generation non-Western background	− 0.11	− 0.25, 0.04	− 0.11	− 0.30, 0.08	− 0.21**	− 0.34, − 0.10	− 0.01	− 0.17, 0.14
Prior education								
University	− 0.41**	− 0.63, − 0.18	0.32**	0.11, 0.52	0.37**	0.21, 0.53	− 0.22	− 0.44, 0.00
Higher vocational education	− 0.22	− 0.74, 0.30	− 0.20	− 0.56, 0.32	− 0.21	− 0.47, 0.06	− 0.70**	− 1.05, − 0.34
Foreign education	1.13**	0.54, 1.72	− 0.03	− 0.83, 0.77	− 0.56**	− 0.91, − 0.22	− 0.61**	− 0.98, − 0.24
Other	− 0.76*	− 1.42, − 0.11	0.38	− 0.42, 1.18	− 0.38	− 0.78, 0.01	− 0.80*	− 1.43, − 0.18
Parental education								
1st generation university applicant	− 0.17*	− 0.30, − 0.03	− 0.12	− 0.31, 0.06	− 0.07	− 0.19, 0.05	− 0.08	− 0.23, 0.07

Pre-GPA = prior education grade point average; CV = curriculum vitae. B refers to the unstandardized regression coefficient together with the 95% confidence interval (95% CI)

*p < .05; **p < .01; ***p < .001

#### Biomedical knowledge test

Biomedical knowledge tests were used by two programs (A and D). Men and applicants who were studying at university-level performed significantly better on biomedical knowledge tests compared to women and applicants from standard pre-university education (respectively, *B* = 0.21, 95% CI [0.06, 0.37]; *B* = 0.32, 95% CI [0.11, 0.52]; Tables 6, 7). Migration background and parental education were not associated with test scores, and the random effect of track was not significant (SD = 0.01, 95% CI [0, 12114,82], *p* = 0.46), indicating that subgroup differences in performance were similar across programs, given the fixed structure considered.

### Performance on broadened criteria

#### Curriculum-sampling test

Three programs included curriculum-sampling tests (A, B, and E). Applicants with a non-Western migration background, both first-generation and second-generation, scored lower on curriculum-sampling tests compared to their traditional counterparts (respectively, *B* = − 0.43, 95% CI [− 0.67, − 0.20]; *B* = − 0.21, 95% CI [− 0.34, − 0.10]; Tables 6, 7). Applicants who were already studying at university-level performed significantly better compared to standard pre-university applicants (*B* = 0.37, 95% CI [0.21, 0.53]), while applicants with foreign education had lower test scores (*B* = − 0.56, 95% CI [− 0.91, − 0.22]). Test scores were not influenced by gender or parental education. Given the fixed structure considered, the random effect of track was significant (SD = 0.09, 95% CI [0.03, 0.32], *p* = 0.01), implying that our overall findings differed between programs. Descriptive statistics of the individual programs employing curriculum-sampling tests (Appendix 1: Table 8) indicate that only for program E, applicants with a first-generation non-Western background had notable low mean Z-scores compared to those without a migration background (M = − 0.86 vs M = 0.29). This difference was smaller for program B (M = − 0.13 vs M = 0.18) and non-existent for program A (M = 0.02 vs M = − 0.01). Noteworthy is that in program E relatively more applicants had a first-generation non-Western background than in the other two programs.

##### CV

Three tracks derived from two different programs included a CV (B, C1, and C2). Compared to women or traditional applicants, CV scores were significantly lower for men (*B* = − 0.17, 95% CI [− 0.31, − 0.02]; Tables 6, 7), first-generation Western immigrants (*B* = − 0.43, 95% CI [− 0.85, − 0.00]), and applicants with higher vocational education, foreign education, and ‘other forms of prior education’ (*B*s between − 0.61 and − 0.81). Parental education was not associated with CV scores. There was a significant effect of track for CV (SD = 0.25, 95% CI [0.08, 0.76] p < 0.001), indicating that the aforementioned effects differed across tracks, given the fixed structure considered. Descriptive statistics (Appendix 1: Table 9) suggest that the gender-based performance gap was smaller for track B compared to the other tracks (track B: M = 0.04 (men) vs M = 0.17 (women); track C1: M = − 0.29 vs M = − 0.06, track C2: M = − 0.11 vs M = 0.25). The overall result that first-generation Western immigrants had lower scores than applicants without a migration background was found for track B (M = − 0.75 vs M = 0.22) and track C2 (M = − 0.42 vs M = 0.09), but not for track C1 (M = − 0.03 vs M = − 0.14). Compared to those without a migration background, applicants with a second-generation non-Western background had lower CV scores in track B (M = − 0.28 vs M = 0.22), similar CV scores in track C1 (M = − 0.17 vs M = − 0.14) and higher CV-scores in track C2 (M = 0.45 vs M = 0.09). For track B, larger differences were observed between different forms of prior education, but this is probably related to the fact that for program C, the tracks were distinguished based on prior education, resulting in a large concentration of standard pre-university education in track C1 and a large concentration of other forms of prior education in track C2.

## Discussion

Unraveling the impact of distinctive selection procedures on student diversity in undergraduate HPE programs requires an insight into how subgroups based on gender, migration background (as an indicator of ethnicity), parental education (as an indicator of SES), and prior education perform on the applied selection tools in different contexts. Our results demonstrated that selection chances of applicants with non-traditional backgrounds were generally smaller, but only significantly for applicants with first-generation Western migration backgrounds and applicants with foreign education. These findings did not differ between programs. However, when taking a closer look, we found larger differences in subgroup performance and more variability in effects. We conclude that the broadened criteria under research—curriculum-sampling tests and CVs—may reduce SES-related performance differences, but not disparities based on applicant ethnicity. Furthermore, subgroup performance differences were context-specific for broadened criteria, but not for traditional criteria.

Our first key finding that the implementation of broadened selection criteria instead of traditional criteria potentially reduces performance disparities based on SES, but may not mitigate an ethnicity-related performance gap, confirms the previous work of Lievens et al. (2016). With respect to the traditional criteria under research, we found that first-generation university applicants had lower pre-GPAs than applicants from traditional backgrounds, also confirming previous research (Griffin & Hu, 2015; Juster et al., 2019; Puddey et al., 2011). Nevertheless, pre-GPAs did not differ between ethnic majority and ethnic minority applicants, which may be explained by a great variety in pre-GPAs between different ethnic minority groups (Puddey et al., 2011). We did not identify significant SES-based and ethnicity-based performance differences on biomedical knowledge tests. However, the sample for this outcome measure was smaller and less diverse compared to the other tools, and international research on such tools persistently reveals such disparities (Girotti et al. 2020; Griffin & Hu, 2015; Juster et al., 2019; Lievens et al., 2016; Puddey et al., 2011). With respect to broadened criteria, our study is the first to investigate subgroup performance on curriculum-sampling tests and CVs in a multi-institutional setting. On both broadened criteria under research, we did not find performance differences based on SES, which resonates with previous research (Griffin & Hu, 2015; Juster et al., 2019; Lievens et al., 2016; Stegers-Jager et al., 2015). Nevertheless, we found that applicants with a migration background were disadvantaged on both tools, whereas previous studies reported mixed findings (Juster et al., 2019; Lievens et al., 2016; Stegers-Jager et al., 2015). A possible explanation for our findings regarding SES is that broadened criteria are less prone to coaching—which is generally more available to high SES applicants (Stemig et al., 2015)—due to their unstandardized and program-specific nature. Traditional criteria, on the other hand, are potentially more susceptible to coaching, as applicants can, for instance, purchase private tutoring to increase their pre-GPA. Simultaneously, the lack of standardization of broadened criteria could increase the risk of cultural bias. Cultural bias can, for instance, occur when certain questions are interpreted differently by members from ethnic minority groups, and may explain the lower scores of applicants with migration backgrounds (Kim & Zebelina, 2015). Language bias probably did not play a significant role in performance disparities based on migration background, because effects were not consistently observed amongst all first-generation immigrants. Additionally, results from previous research suggest that disparities also exist for immigrants from Dutch-speaking countries (Stegers-Jager et al., 2015).

A second key finding is that for the broadened selection criteria, subgroup performance differences were context-specific, whereas the traditional selection criteria had consistent effects across programs. This is in accordance with the current evidence for subgroup differences in performance on the two types of criteria: results from prior research regarding the use of broadened criteria are mixed (Juster et al., 2019; Lievens et al., 2016; Stegers-Jager, 2018; Stegers-Jager et al., 2015), while the outcomes from traditional criteria are rather consistent in disadvantaging ethnic minority and lower SES applicants (Griffin & Hu, 2015; Juster et al., 2019; Lievens et al., 2016; Puddey et al., 2011; Stegers-Jager et al., 2015). Additionally, the overall finding that men and applicants with a first-generation Western migration background had lower CV scores was not in line with a previous Dutch single-institution study (Stegers-Jager et al., 2015). Our study is the first to directly demonstrate that seemingly comparable tools can have differential effects on subgroup performance across different programs. Typically, broadened criteria allow for more variation and can be further adjusted to the specific program contents, which may be the cause of stronger context-dependent effects on subgroup performance for these tools. For instance, curriculum-sampling tests vary in their subject, preparatory materials, and preparation time. Additionally, previous research suggests that the complexity of the language (Lievens et al., 2016), and question format (Edwards & Arthur, 2007), may contribute to subgroup differences in test performance. Likewise, the scoring method and the type of extracurricular activities that are considered in CV scores may play a role, since healthcare experiences are considered to be unequally accessible to applicants from different backgrounds (Wouters, Croiset, Isik, et al., 2017).

A third key finding is that subgroup differences in performance on individual tools did not always have consequences for the probability of selection of those subgroups. We found that selection chances were only significantly smaller for applicants with a first-generation Western migration background and applicants with foreign education, two subgroups that were left unnoticed by previous research. Combining tools with differential subgroup performance within procedures and across procedures may have counter-balanced the overall effect, and the weightings of different tools may have played a role (Lievens et al., 2016; Stegers-Jager, 2018). Our findings are not fully supported by the results from a recent retrospective study that included applicants to all Dutch undergraduate HPE programs (Mulder et al., 2022). The authors found significantly lower selection probability for additional ethnic minority groups, men and lower SES groups, although the results were negligible in terms of statistical effect size (Chen et al., 2010). The discrepancy between findings may be explained by differences between target groups: the present study used prospective data of a subset of programs and included a more heterogenous group of applicants, including older applicants and those with foreign education.

Strengths of our study include that we collected data from multiple programs and that we used a multilevel analytical approach, creating the opportunity to correct for and examine contextual differences. The typical Dutch admissions system, which allows schools to design their own selection procedure, allowed us to compare a variety of (applications of) tools. As a consequence, not all tools were used by all programs. Therefore, direct comparison across different outcome measures and examination of the correlation of performance between different tools were not possible. Another limitation is that although the present study is, to our knowledge, the first to include the selection procedures of a range of different types of undergraduate HPE programs, it was not possible to cover all specialties and institutions. This may have consequences for the generalizability of our findings. Furthermore, we included parental education as a relevant indicator for SES, since first-generation university applicants have been shown to face barriers during the transition into higher education (Stephens et al., 2014), but we may have overlooked other potentially relevant SES-related effects, such as parental income and profession (Girotti et al. 2020; Mulder et al., 2022; Steven et al., 2016). Likewise, migration background is a stable and objective indicator of ethnicity, but does not account for ethnic identity (Ross et al., 2020; Stronks et al., 2009). Another limitation is that for certain subgroups, sample sizes were small, thus those results should be interpreted with caution. Finally, two selection procedures were partly affected by COVID-19 measures, potentially reducing the generalizability of our findings. Nevertheless, the effects of the probability of selection did not differ significantly between the four programs in that analysis, of which two were affected by COVID.

The variety in subgroup differences between and within tools implies that future research should determine whether specific characteristics of tools can play a moderating role in their effects on diversity. This could lead to the identification of best practices. Furthermore, based on our results we cannot draw conclusions with respect to the effect of different weightings of tools. Therefore, we endorse a previous suggestion to investigate the effects of different weightings of tools on student diversity (Stegers-Jager, 2018). Future studies should also examine whether selection tools differentially predict academic performance for different subgroups, to determine whether the performance disparities we found correspond with bias (i.e., underprediction or overprediction for certain subgroups). Finally, future research should identify the specific underlying characteristics and needs of subgroups of applicants with non-traditional backgrounds within the context of HPE selection, to provide better support during and, as suggested by others (Lievens, 2015; Wouters, 2020), also after selection. For instance, applicants from alternative forms of prior education may face difficulties managing the expectations in HPE selection that can strongly differ from their previous educational experiences (Katartzi & Hayward, 2020; Rienties & Tempelaar, 2013).

From a practical viewpoint, the context-specificity of subgroup differences in performance indicates that HPE programs need to establish continuous evaluation of the possible effects of their selection procedures on student diversity, rather than only relying on existing research in other contexts. Additionally, we encourage programs to conscientiously include and/or develop alternative tools that can reduce adverse impact and explicitly promote well-needed diversity, such as SJTs (Juster et al., 2019) and multiple mini-interviews (Griffin & Hu, 2015), while keeping in mind that effects can be context-specific. We acknowledge the desire to apply school-specific selection procedures, as selection procedures that align their contents with the curriculum can have high predictive value (Schreurs et al., 2020). Simultaneously, this creates a responsibility for programs to evaluate different aspects of the validity of their selection procedure, including adverse impact (Schreurs, 2020). Additionally, programs could consider validating their tools with diverse norming groups (Padilla & Borsato, 2008).

In conclusion, selection into undergraduate HPE programs can unintentionally impact student diversity, hindering equitable admission. Compared to traditional criteria, broadened criteria can reduce SES-related performance differences, but not disparities based on ethnicity. For broadened criteria, subgroup differences in performance also vary across contexts. We, therefore, call for continuous evaluation effects of selection on diversity, the identification of best practices within existing tools, the inclusion of tools with a positive or neutral impact on student diversity, and sufficient quality control.

## Appendix

See Tables 6, 7, 8 and 9.

**Table 6:** Characteristics of 1935 applicants to five HPE undergraduate programs

	A (N = 505, RR = 59%)^a^			B (N = 640, RR = 74%)^a^			C (N = 305, RR = 34%)^a^			D (N = 241, RR = 81%)^b^			E (N = 246, RR = 67%)^a,c^		
	N (%) applicant pool	N (%) selected applicants	% Selected	N (%) applicant pool	N (%) selected applicants	% Selected	N (%) applicant pool	N (%) selected applicants	% Selected	N (%) applicant pool	N (%) selected applicants	% Selected	N (%) applicant pool	N (%) selected applicants	% Selected
Gender															
Woman	332 (66)	141 (64)	43	443 (70)	214 (72)	48	230 (75)	116 (81)	50	163 (67)	84 (67)	52	179 (73)	170 (72)	95
Man	173 (34)	81 (37)	47	194 (30)	83 (28)	43	75 (25)	28 (19)	37	78 (33)	41 (33)	52	67 (27)	66 (28)	99
Migration background															
No migration background	324 (64)	256 (65)	45	380 (60)	194 (65)	51	169 (68)	81 (69)	48	211 (87)	112 (89)	53	117 (47)	116 (49)	99
1st generation Western background	6 (1)	2 (< 1)	33	25 (4)	6 (2)	32	9 (3)	1 (< 1)	11	3 (1)	1 (< 1)	33	2 (< 1)	2 (< 1)	100
2nd generation Western background	37 (7)	22 (10)	60	45 (7)	20 (7)	44	24 (8)	12 (8)	50	15 (6)	8 (6)	53	9 (3)	9 (4)	100
1st generation non-Western background	16 (3)	7 (3)	44	33 (5)	12 (4)	36	24 (8)	10 (7)	42	2 (1)	0 (0)	0	33 (13)	24 (10)	73
2nd generation non-Western background	122 (24)	46 (21)	38	154 (24)	65 (22)	42	79 (26)	40 (28)	51	12 (5)	6 (5)	50	85 (32)	85 (36)	100
Prior education															
Pre-university education	375 (74)	148 (67)	40	529 (83)	268 (90)	51	209 (68)	114 (79)	55	228 (94)	120 (95)	53	202 (82)	200 (84)	99
University	100 (20)	60 (27)	60	48 (8)	19 (6)	40	67 (22)	22 (15)	33	10 (4)	4 (3)	40	11 (5)	11 (5)	100
Higher vocational education	19 (4)	9 (4)	47	21 (3)	6 (2)	29	12 (4)	3 (2)	25	2 (1)	1 (1)	50	14 (6)	12 (5)	86
Foreign education	6 (1)	3 (1)	50	32 (5)	4 (1)	13	11 (4)	4 (3)	36	1 (< 1)	1 (1)	100	4 (2)	2 (1)	50
Other	5 (1)	2 (1)	40	5 (1)	0 (0)	0	6 (2)	1 (1)	17	1 (< 1)	0 (0)	0	15 (6)	11 (5)	73
Parental education															
No 1st generation university applicant	385 (76)	167 (75)	43	473 (75)	231 (78)	49	207 (68)	103 (72)	50	205 (85)	111 (88)	54	175 (71)	169 (72)	97
1st generation university applicant	119 (24)	55 (25)	46	160 (25)	66 (22)	41	96 (32)	41 (28)	43	37 (15)	15 (12)	41	71 (29)	67 (28)	94

N = number of individuals; RR = response rate

^a^Located in an urban area

^b^Located in a rural area

^c^Results were excluded from multilevel logistic analysis for probability of selection

**Table 7:** Applicant characteristics by track for program C

	C total (N = 305)	C1 (N = 175)	C2 (N = 130)
	N (%)	N (%)	N (%)
Gender			
Woman	230 (75)	136 (78)	94 (72)
Man	75 (25)	39 (22)	36 (28)
Migration background			
No migration background	169 (68)	101 (58)	68 (52)
1st generation Western background	9 (3)	3 (2)	6 (5)
2nd generation Western background	24 (8)	18 (10)	6 (5)
1st generation non-Western background	24 (8)	9 (5)	15 (12)
2nd generation non-Western background	79 (26)	44 (25)	35 (27)
Prior education			
Pre-university education	209 (68)	171(98)	38 (29)
University	67 (22)	0 (0)	67 (52)
Higher vocational education	12 (4)	0 (0)	12 (9)
Foreign education	11 (4)	4 (2)	7 (5)
Other	6 (2)	0 (0)	6 (5)
Parental education			
No 1st generation university applicant	207 (68)	122 (70)	85 (65)
1st generation university applicant	96 (32)	52 (30)	44 (34)

N = number of individuals

**Table 8:** Descriptive statistics of Z-scores on curriculum-sampling tests for applicant subgroups by program

	Program A			Program B			Program E		
	N	M	SD	N	M	SD	N	M	SD
Gender									
Woman	331	− 0.03	0.92	392	0.10	0.90	179	− 0.01	0.98
Man	173	− 0.02	1.04	173	− 0.02	1.09	67	0.03	1.07
Migration background									
No migration background	323	− 0.01	0.95	332	0.18	0.85	117	0.29	0.90
1st generation Western background	6	− 0.06	0.74	22	− 0.13	1.07	2	− 0.67	0.00
2nd generation Western background	37	0.28	0.71	40	0.01	1.27	9	0.09	0.84
1st generation non-Western background	16	0.02	1.07	29	− 0.07	1.00	33	− 0.86	1.02
2nd generation non-Western background	122	− 0.16	1.02	142	− 0.13	1.08	85	− 0.07	0.96
Prior education									
Pre-university education	374	− 0.10	0.98	459	0.06	0.94	202	0.10	0.95
University	100	0.27	0.81	48	0.48	0.73	11	0.54	− 0.92
Higher vocational education	19	0.06	1.00	21	− 0.04	1.43	14	− 0.85	1.16
Foreign education	6	− 0.53	1.16	30	− 0.46	1.11	4	− 1.07	0.67
Other	5	− 0.48	0.36	5	0.14	0.48	15	− 0.71	0.78
Parental education									
No 1st generation university applicant	384	− 0.03	0.98	411	0.12	0.90	175	0.08	0.93
1st generation university applicant	119	− 0.00	0.81	150	− 0.06	1.13	71	− 0.19	1.14

N = number of individuals; M = mean; SD = standard deviation. Although we use Z-scores, the overall mean can deviate slightly from 0, and the overall SD can deviate slightly from 1, because we only used data from applicants who provided consent, thus not all selection outcomes, on which the Z-scores are based, are included

**Table 9:** Descriptive statistics of Z-scores on curriculum vitae for applicant subgroups by track

	Track B			Track C1			Track C2		
	N	M	SD	N	M	SD	N	M	SD
Gender									
Woman	392	0.17	0.99	139	− 0.06	1.00	94	0.25	1.05
Man	173	0.04	1.03	39	− 0.29	0.84	36	− 0.11	0.91
Migration background									
No migration background	332	0.22	0.96	101	− 0.14	0.93	68	0.09	1.03
1st generation Western background	22	− 0.75	0.86	3	− 0.03	0.89	6	− 0.42	0.39
2nd generation Western background	40	0.24	1.16	18	− 0.29	0.68	6	− 0.20	0.55
1st generation non-Western background	29	0.24	1.16	9	0.83	1.50	16	0.08	1.08
2nd generation non-Western background	142	− 0.28	0.93	44	− 0.17	0.97	35	0.45	1.09
Prior education									
Pre-university education	459	0.26	0.96	171	− 0.13	0.96	38	0.24	1.17
University	48	− 0.15	1.05	0			67	0.26	0.97
Higher vocational education	21	− 0.48	0.74	0			12	− 0.28	0.95
Foreign education	30	− 0.80	0.84	4	0.56	1.38	7	− 0.27	0.84
Other	5	− 0.98	0.69	0			6	− 0.31	0.72
Parental education									
No 1st generation university applicant	411	0.16	1.02	122	− 0.12	0.94	85	0.16	1.01
1st generation university applicant	150	0.06	0.95	52	− 0.08	1.03	44	0.14	1.07

N = number of individuals; M = mean; SD = standard deviation. Although we use Z-scores, the overall mean can deviate slightly from 0, and the overall SD can deviate slightly from 1, because we only used data from applicants who provided consent, thus not all selection outcomes, on which the Z-scores are based, are included
